# Enhancing Road Sensor Data Fidelity via FMD Decomposition for Accurate Traffic Flow Prediction Using EBWO-Optimized LGC-BiGRU

**DOI:** 10.3390/s26154813

**Published:** 2026-07-29

**Authors:** Jixiao Jiang, Anastasia Feofilova, Ivan Topilin, Nikita Beskopylny

**Affiliations:** Department of Transportation and Traffic Management, Don State Technical University, 344002 Rostov-on-Don, Russia; s2387788@stud.donstu.ru (J.J.); itopilin@donstu.ru (I.T.); nbeskopylnyi@donstu.ru (N.B.)

**Keywords:** sensor data processing, traffic flow prediction, deep learning framework, optimization algorithm, intelligent transportation system

## Abstract

**Highlights:**

**What are the main findings?**

- We introduce an adaptive frequency domain segmentation mechanism into the FMD method. This mechanism significantly outperforms traditional decomposition techniques (such as EMD and VMD) in denoising raw road sensor data, effectively separating non-stationary noise and outliers to achieve higher prediction accuracy.
- The FMD-EBWO-LGC-BiGRU framework achieves efficient denoising of road sensor data and traffic flow prediction. The improved EBWO adaptively determines the key hyperparameters of the LGC-BiGRU model, eliminating the need for traditional empirical parameter tuning.

**What are the implications of the main findings?**

- The improved EBWO provides a more efficient metaheuristic optimizer for tuning deep learning models in high-dimensional traffic perception environments. It improves the model’s generalization ability while reducing manual tuning workload.
- The combination of EBWO and FMD-LGC-BiGRU constructs a complete workflow from raw sensor data augmentation for accurate traffic flow prediction. This provides a feasible application solution for resource-constrained ITSs.

**Abstract:**

In resource-constrained intelligent transportation systems (ITSs), raw road sensor data is often affected by non-stationary noise and outliers, which severely reduces the accuracy of traffic flow prediction. To address this, this paper proposes a hybrid prediction framework that integrates Frequency Mode Decomposition (FMD), Enhanced Beluga Whale Optimization (EBWO), and the Logistic-Gaussian Circle-based Bidirectional Gated Recurrent Unit (LGC-BiGRU). The proposed FMD module introduces an adaptive frequency domain segmentation mechanism, which effectively separates noise from the data without introducing modal aliasing. EBWO employs an adaptive balancing factor mechanism to dynamically explore the spatiotemporal characteristics of traffic flow, preventing the framework from getting trapped in local optima. For predictive modeling, the LGC-BiGRU integrates an LGC Optimizer, mapping features to a circular probability space to enhance sensitivity to periodic patterns. Experimental results show that, compared with the best-performing state-of-the-art model, our framework reduces the mean absolute error (MAE) by an average of 30.6% and the root mean square error (RMSE) by 29.9%. These results confirm the framework’s effectiveness and feasibility for traffic flow prediction under complex noise.

## 1. Introduction

With the rapid deployment of roadside sensors and loop detectors in the road network, the continuous collection of large amounts of traffic data provides a data foundation for traffic flow prediction [1,2,3]. However, the reliability of this data is often affected by hardware failures and communication instability, introducing non-stationary noise and outliers [4,5]. These noises and outliers propagate along the prediction process, ultimately leading to significant prediction errors [6]. This error propagation phenomenon severely limits the accuracy of traffic flow prediction in ITSs, thereby affecting the reliability of applications such as traffic scheduling, route planning, and congestion warning [7,8]. This challenge is not unique to road traffic: in the railway domain, similar issues of sensor data reliability and system validation under uncertain conditions have been extensively studied, particularly in the context of obstacle detection, track intrusion detection systems, and virtual environment validation of traffic management modules [9,10]. Therefore, to ensure the reliable operation of ITSs, the traffic flow prediction process must address data preprocessing, traffic flow feature extraction, and model optimization to collaboratively suppress noise propagation and ensure prediction accuracy [11].

Numerous studies have improved the accuracy of traffic flow prediction through advanced time modeling and data preprocessing techniques. In temporal modeling, Bidirectional Long Short-Term Memory (BiLSTM) networks and Bidirectional Gated Recurrent Units (BiGRUs) have been widely used to capture the sequential dependencies in traffic data [12,13]. In recent years, Zeng et al. verified the superiority of GRUs in traffic flow prediction and proposed a CSC-GRU model that incorporates spatial correlations [14]. Guo et al.’s DSTSFN directly learns the dependencies between source and target nodes using a bipartite graph, achieving a 2.27% accuracy improvement with only 8.98% of the training time of the state-of-the-art model [15]. Wang et al., on the other hand, achieved accurate predictions by modeling both static and dynamic spatial dependencies using an HSTGNN that integrates attention mechanisms [16]. The MGTEFormer model developed by Huang et al. employs a multi-granularity temporal embedding fusion strategy to integrate features from different time granularities into historical traffic flow data, effectively reducing the loss rate of predicted data [17]. Bao et al.’s DA-RMN model, combining a denoising diffusion model with a gated attention fusion mechanism, effectively enhances the efficiency of key traffic flow feature extraction [18]. Liu and Meidani proposed a heterogeneous graph neural network for traffic assignment, which achieves faster convergence and higher accuracy by explicitly modeling different types of intersections and road segments in urban traffic networks [19]. While these advancements have improved the accuracy of traffic flow prediction, the performance of these models is fundamentally limited by the quality of the input data. When the original sensor measurement data is disturbed by noise, the models often overfit spurious fluctuations and fail to capture true traffic dynamics.

For handling the noise and erroneous signals carried by sensor data, signal decomposition is a promising technical approach. Methods such as Empirical Mode Decomposition (EMD) and Variational Mode Decomposition (VMD) can separate raw traffic signals into different intrinsic mode functions (IMFs), thus separating noise components from traffic characteristics [20]. For example, Bing et al. proposed a short-term traffic flow prediction method based on CEEMDAN-VMD quadratic decomposition, which performs a quadratic decomposition of the highest-frequency IMF1 component to preserve traffic flow characteristics, achieving a 22% reduction in the MAPE index compared to single-decomposition methods [21]. Bie et al., for satellite sensor traffic data, employed VMD combined with a CNN-BiLSTM model, effectively improving the prediction robustness of non-stationary traffic sequences [22]. Lu et al. also used VMD combined with GRU for prediction and verified the effectiveness of the decomposition strategy on three real sensor datasets [23]. Furthermore, Shen and Zhang proposed an EMD method for multivariate traffic time series, which effectively reduces computational burden and improves adaptability by defining an envelope function [24]. However, in practical prediction, the EMD method faces the problem of aliasing of different IMFs [25,26]. VMD, on the other hand, requires pre-determining the number of IMFs, and the model parameters change with the number of IMFs [27,28]. These challenges collectively limit the application of existing decomposition methods in ITSs.

Despite these efforts, significant research gaps remain: even state-of-the-art deep learning models are fundamentally limited by the quality of sensor data. This gap is particularly pronounced in resource-constrained ITS environments, where sensor failures and communication instability are common. Furthermore, deep learning models for traffic flow prediction typically involve a large number of hyperparameters. The optimal values for these hyperparameters vary depending on the dataset, and manually determining these optimal values is extremely difficult. Currently, no framework can simultaneously address adaptive data denoising, automatic hyperparameter optimization, and enhanced sensitivity to periodic patterns.

To overcome these limitations, this paper proposes an integrated framework called FMD-EBWO-LGC-BiGRU. The main contributions of this paper are threefold:

1. We introduce an FMD module with an adaptive frequency domain segmentation mechanism. It can automatically divide the frequency bands according to the energy spectrum distribution of the signal, thereby avoiding mode aliasing. This module can effectively separate non-stationary noise and outliers from raw sensor data without manually specifying the number of modes.

2. We propose an enhanced EBWO. This optimizer uses a tent chaotic map for population initialization, employs an adaptive balance factor to dynamically coordinate exploration and development, and uses an improved Lévy flight strategy for adaptive step size adjustment. This enables automatic optimization of key hyperparameters in the LGC-BiGRU model, eliminating the need for tedious manual tuning.

3. We design a BiGRU module based on a logistic Gaussian circle. This module first projects traffic features onto a circular probability space using an LGC map to enhance sensitivity to periodic patterns, and then employs two stacked BiGRU layers to capture bidirectional temporal dependencies. This design enables the framework to comprehensively capture the nonlinear evolution of traffic flow data.

It should be noted that the proposed framework is designed as a time-based predictive model, focusing on modeling the temporal dynamics of individual traffic sensors rather than capturing the spatial correlations between multiple sensors. This design makes the framework particularly suitable for scenarios where road network topology is unavailable, which aligns with our motivation to address data quality challenges in such environments.

## 2. Materials and Methods

**Figure 1:**
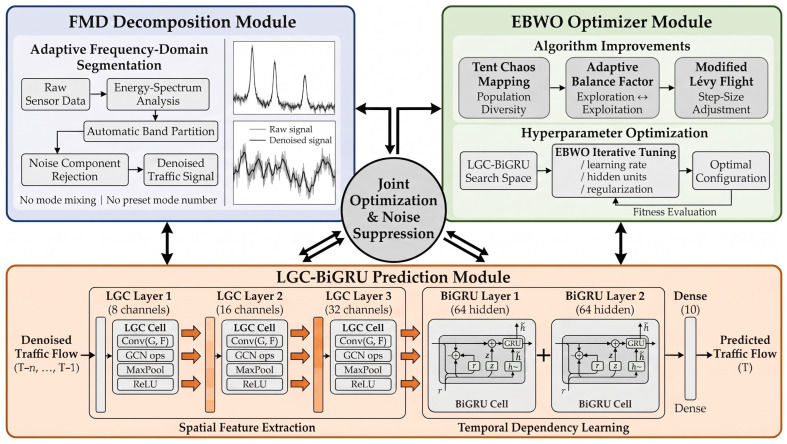
Overall architecture of the FMD-EBWO-LGC-BiGRU framework.

The overall architecture of the FMD-EBWO-LGC-BiGRU framework is shown in Figure 1. It adopts a triangular closed-loop structure, consisting of three collaborative modules surrounding a joint optimization node.

In this framework, the FMD module receives raw sensor data, performs adaptive frequency domain segmentation based on the signal energy spectrum distribution, and outputs the denoised traffic signal. The EBWO module integrates three algorithm enhancement mechanisms: tent chaos mapping to ensure population diversity, an adaptive balance factor to coordinate exploration and exploitation, and an improved Lévy flight to achieve adaptive step size. These mechanisms enable EBWO to iteratively predict the optimal hyperparameters of the network and feed the output back to the optimization process through a fitness evaluation feedback loop. The LGC-BiGRU prediction module constructs a hierarchical feature representation through three Logistic-Gauss circular layers, followed by two stacked BiGRU layers to capture bidirectional temporal dependencies. The three modules work collaboratively in a closed-loop manner. The high-quality data provided by FMD supports the optimization process of EBWO; the optimal hyperparameters output by EBWO directly configure LGC-BiGRU; and the prediction errors generated by LGC-BiGRU are propagated back to FMD to adjust its decomposition strategy. This closed-loop collaborative relationship enables the three modules to converge to the “joint optimization and noise suppression” objective identified by the central node, which is different from the sequential pipeline structure of the traditional framework.

### 2.1. FMD Module Modeling

**Figure 2:**
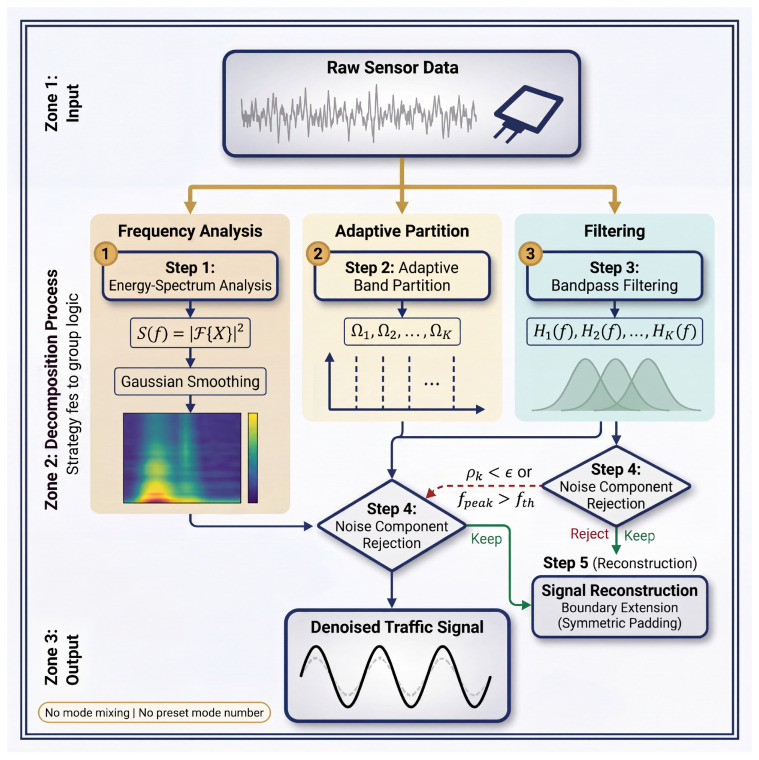
FMD module operation process.

As shown in Figure 2, the FMD module follows a sequential workflow comprising five core steps: energy spectrum analysis, adaptive frequency band division, bandpass filtering, noise component suppression, and signal reconstruction. Unlike traditional decomposition methods, the proposed FMD module can operate without recursive filtering or pre-setting the number of modes.

Figure 2 illustrates the transformation process of the raw sensor data in five steps, with arrows indicating the forward propagation of information. The energy spectrum analysis step first transforms the input signal to the frequency domain and calculates its power spectral density. The adaptive bandgap step detects local minima in the smoothed spectrum, dividing the frequency axis into data-driven, non-overlapping bands. Subsequently, the bandpass filtering step extracts the individual IMFs corresponding to each band. The noise component suppression step evaluates each mode using two criteria: energy contribution rate and peak frequency. Finally, the signal reconstruction step sums the retained modes to generate the denoised output.

The mathematical formulation of the FMD module is presented below. Let the raw sensor data sequence be denoted as $X=\left\{x_{1},x_{2},\ldots ,x_{T}\right\}\in R^{T}$, where *T* is the length of the time window. The objective of the FMD module is to recover the potential clean traffic signal *s*(*t*) from the noisy observation $x_{t}=s\left(t\right)+\eta \left(t\right)+\delta \left(t\right)$, with *η*(*t*) representing non-stationary noise and *δ*(*t*) representing sparse outliers. To achieve this, the module first applies a discrete Fourier transform to convert the time-domain signal into the frequency domain:

(1) $$X\left(f\right)=\sum_{t=0}^{T-1}x\left(t\right)\cdot e^{\frac{-j2\pi ft}{T}},f=0,1,\ldots ,T-1,$$

where *t* is the time index, *f* is the frequency index, *j* is the imaginary unit, and *X*(*f*) is the complex Fourier coefficient at frequency *f*.

The energy distribution across frequencies is then given by the squared amplitude:

(2) $$P\left(f\right)={\left|X\left(f\right)\right|}^{2},$$

where *P*(*f*) is the power spectral density, representing how much energy the signal contains at frequency *f*.

To avoid over-segmentation caused by noisy spectral fluctuations, a Gaussian smoothing kernel is applied. Let m be an integer offset relative to the current frequency index *f*, ranging from −M to M, where M defines the half-width of the smoothing window. The smoothed spectrum is then computed as:

(3) $$\tilde{P}\left(f\right)=\sum_{m=-M}^{M}P\left(f+m\right)\cdot g\left(m\right),$$

where $g\left(m\right)=\frac{1}{\sqrt{2\pi }\sigma }e^{-m^{2}/\left(2\sigma^{2}\right)}$ is the Gaussian kernel with standard deviation *σ*. The term $P\left(f+m\right)$ denotes the original power spectral density at frequency index *f* + *m*, and the summation performs a weighted average of neighboring frequency bins.

After obtaining the smoothed spectrum $\tilde{P}\left(f\right)$, the module locates the boundaries between frequency bands. A boundary is identified at frequency index *f* where $\tilde{P}\left(f\right)$ is a local minimum. Specifically, the condition for a local minimum is:

(4) $$\tilde{P}\left(f-1\right)>\tilde{P}\left(f\right)\;\mathrm{and}\;\tilde{P}\left(f\right)<\tilde{P}\left(f+1\right),$$

Let the detected boundary indices be denoted as $f_{0},f_{1},\ldots ,f_{K}$, sorted in ascending order, with $f_{0}=0$ and $f_{K}=\left\lfloor T/2\right\rfloor$. The *i*-th frequency band is then defined as:

(5) $$\Omega_{i}=\left\{f\left|f_{i-1}\leq f<f_{i}\right.\right\},i=1,2,\ldots ,K,$$

where *K* is the total number of bands determined automatically from the data.

For each band $\Omega_{i}$, a bandpass filter with smooth roll-off characteristics is constructed. The filter response at frequency *f* is defined as:

(6) $$Y_{i}\left(f\right)=X\left(f\right)\cdot H_{i}\left(f\right),$$

where *X*(*f*) is the Fourier coefficient at frequency *f*, and $H_{i}\left(f\right)$ is the filter response for band *i*. The product gives $Y_{i}\left(f\right)$, the filtered spectrum containing only frequency components within band *i*.

The time-domain representation of each band, referred to as the *i*-th IMF, is obtained via the inverse discrete Fourier transform:

(7) $$y_{i}\left(t\right)=\frac{1}{T}\sum_{f=0}^{T-1}Y_{i}\left(f\right)\cdot e^{\frac{j2\pi ft}{T}},$$

where $y_{i}\left(t\right)$ is the oscillatory signal of band *i* at time *t*.

To identify noise-dominated IMFs, two criteria need to be evaluated. The first criterion is the energy contribution rate:

(8) $$r_{i}=\frac{\sum_{t=0}^{T-1}{\left|y_{i}\left(t\right)\right|}^{2}}{\sum_{t=0}^{T-1}{\left|x\left(t\right)\right|}^{2}},$$

where $r_{i}$ represents the proportion of the total signal energy contained in the *i*-th mode.

The second is the peak frequency:

(9) $$f_{i}^{\mathrm{peak}}=\arg\underset{f}{\max}\left|Y_{i}\left(f\right)\right|,$$

A mode is classified as noise and rejected if:

(10) $$r_{i}<\varepsilon \;\wedge \;f_{i}^{\mathrm{peak}}>f_{\mathrm{high}},$$

where ε is the energy threshold and $f_{\mathrm{high}}$ is the high-frequency cutoff frequency.

The denoised traffic signal $\hat{s}\left(t\right)$ is then reconstructed by summing the retained IMFs:

(11) $$\hat{s}\left(t\right)=\sum_{i\notin R}y_{i}\left(t\right),\;R=r_{i}<\varepsilon \;\wedge \;f_{i}^{\mathrm{peak}}>f_{\mathrm{high}}\;,$$

To reduce boundary artifacts, a symmetric filling strategy is applied before decomposition. The complete operation can be represented as:

(12) $$\mathrm{FMD}\left(x\right)=\sum_{i\notin R}F^{-1}\left\{F\left\{x\right\}\cdot H_{i}\right\},$$

where *F* is the Fourier transform, $F^{-1}$ is the inverse Fourier transform, and FMD(*x*) is the output of the entire decomposition and denoising process applied to the input signal *x*.

These formulas in the FMD module achieve three main advantages: data-driven band selection, strict mode orthogonality, and noise suppression. These three points distinguish FMD from traditional methods such as EMD and VMD.

### 2.2. EBWO Module Modeling

**Figure 3:**
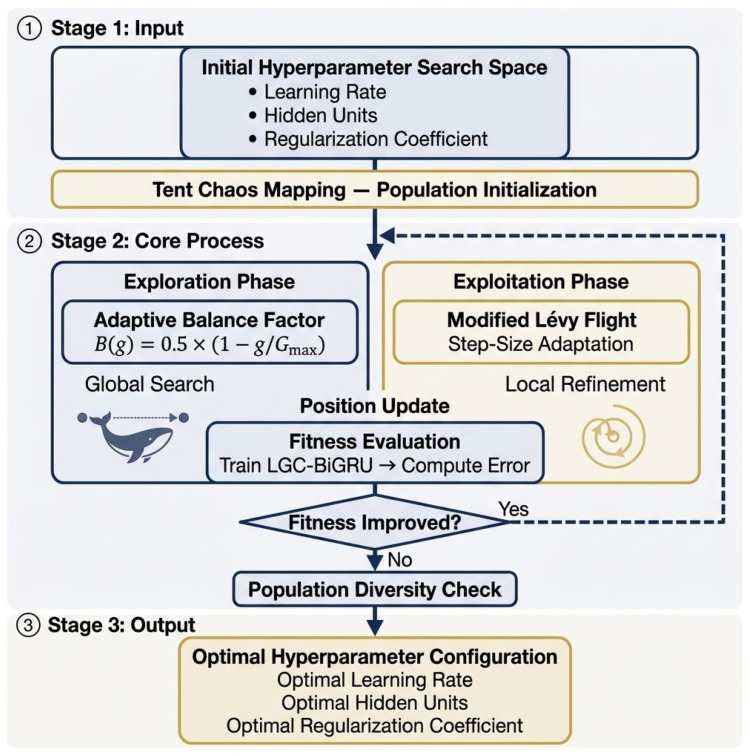
Iterative evolution process of the EBWO module.

The EBWO module optimizes the hyperparameters of the LGC-BiGRU prediction model through an iterative evolutionary process. Its workflow follows a closed-loop structure (Figure 3), comprising five sequential stages: population initialization, adaptive balance control, exploration phase, development phase, and fitness evaluation and termination check.

The EBWO process begins with the initialization phase. In this phase, the Tent chaotic map generates a diverse set of hyperparameter configurations. Each configuration represents a position of a whale in the search space and is evaluated by training the LGC-BiGRU model and calculating its prediction error. Subsequently, an adaptive balancing factor determines whether the algorithm explores new regions or utilizes existing promising regions. During the exploration phase, the whale performs a global search with a large step size to avoid getting trapped in local optima. During the utilization phase, an improved Lévy fly algorithm optimizes candidate solutions around the current best configuration. After each position update, the fitness of the new configuration is evaluated. If the fitness improves, the best configuration is updated; otherwise, population diversity is checked. If the diversity is below a threshold, the population is reinitialized using the tent chaotic map to avoid stagnation. The loop terminates when the maximum number of iterations is reached or the fitness does not significantly improve.

Let *N* be the population size and *D* be the number of hyperparameters to be optimized. Each candidate configuration is denoted as $\theta =\left(\theta_{1},\theta_{2},\ldots ,\theta_{D}\right)$, and its fitness is measured by the prediction error of the LGC-BiGRU model on the validation set, denoted as *F*(*θ*).

To generate an initial population with good diversity, EBWO employs Tent chaos mapping instead of random initialization. The Tent map produces a chaotic sequence as follows:

(13) $$z_{p+1}=\left\{\begin{array}{cc} 2z_{p}, & 0\leq z_{p}<0.5\\ 2\left(1-z_{p}\right), & 0.5\leq z_{p}<1 \end{array}\right.,$$

where $m_{p}\in \left(0,1\right)$ is the *p*-th chaotic state.

The initial value of the *d*-th hyperparameter can be obtained by mapping the chaotic state to the search boundary:

(14) $$\theta_{d}^{\left(0\right)}=L_{d}+z_{d}\cdot \left(U_{d}-L_{d}\right),$$

where $L_{d}$ and $U_{d}$ are the lower and upper bounds of the *d*-th hyperparameter. Compared to random sampling, this chaotic initialization can distribute candidate configurations more evenly in the search space, thus preventing the predictive model from converging prematurely.

Once the population is initialized, EBWO enters its main optimization loop. At each generation *g*, the algorithm uses an adaptive balance factor $B^{\left(g\right)}$ to switch between global exploration and local exploitation. This factor is defined as:

(15) $$B^{\left(g\right)}=0.5\cdot \left(1-\frac{g}{G_{\max}}\right),$$

where *g* is the current iteration number and $G_{\max}$ is the maximum iteration number. The factor $B^{\left(g\right)}$ has an initial value of approximately 0.5 and decreases linearly to 0. In the early stages of iteration, the value of $B^{\left(g\right)}$ is relatively large, which is beneficial for exploration. As the algorithm converges to the optimal solution, the value of $B^{\left(g\right)}$ gradually decreases to 0, which is beneficial for local fine-tuning near the current optimal solution.

When the algorithm enters the development phase, EBWO employs an improved Lévy flight to optimize candidate solutions around the current optimal configuration. Its position update equation is expressed as:

(16) $$\theta^{\left(g+1\right)}=\theta^{\left(g\right)}+\alpha \cdot \mathrm{Levy}\left(\lambda \right)⊙\left(\theta_{\mathrm{best}}-\theta^{\left(g\right)}\right),$$

where $\theta_{\mathrm{best}}$ is the best configuration found so far, $\mathrm{Levy}\left(\lambda \right)$ generates a random step from a Lévy distribution with exponent λ, and *α* is an adaptive scaling factor:

(17) $$\alpha =\alpha_{0}\cdot \left(1-\frac{F\left(\theta \right)}{F_{\max}}\right),$$

where $\alpha_{0}$ is the initial step size, $F\left(\theta \right)$ is the fitness of the current configuration, and $F_{\max}$ is the maximum fitness in the current population. This mechanism automatically reduces the step size when the configuration already achieves good fitness, allowing finer local search near the optimum.

To prevent premature convergence caused by loss of population diversity, EBWO monitors the spread of candidate configurations at each generation. The population diversity at generation *g*, denoted as Div^(*g*)^, is defined as:

(18) $${\mathrm{Div}}^{\left(g\right)}=\frac{1}{N}\sum_{r=1}^{N}\sqrt{\sum_{d=1}^{D}{\left(\frac{\theta_{r,d}^{\left(g\right)}-{\bar{\theta }}_{d}^{\left(g\right)}}{U_{d}-L_{d}}\right)}^{2}},$$

where ${\bar{\theta }}_{d}^{\left(g\right)}$ is the mean value of the *d*-th hyperparameter across all whales at generation *g*, and $\theta_{r,d}^{\left(g\right)}$ is the value of the *d*-th hyperparameter for the *r*-th whale at generation *g*.

The optimization loop terminates when EBWO reaches the maximum number of iterations $G_{\max}$. The optimal hyperparameter configuration found by EBWO is then used to configure the LGC-BiGRU prediction module.

### 2.3. LGC-BiGRU Module Modeling

**Figure 4:**
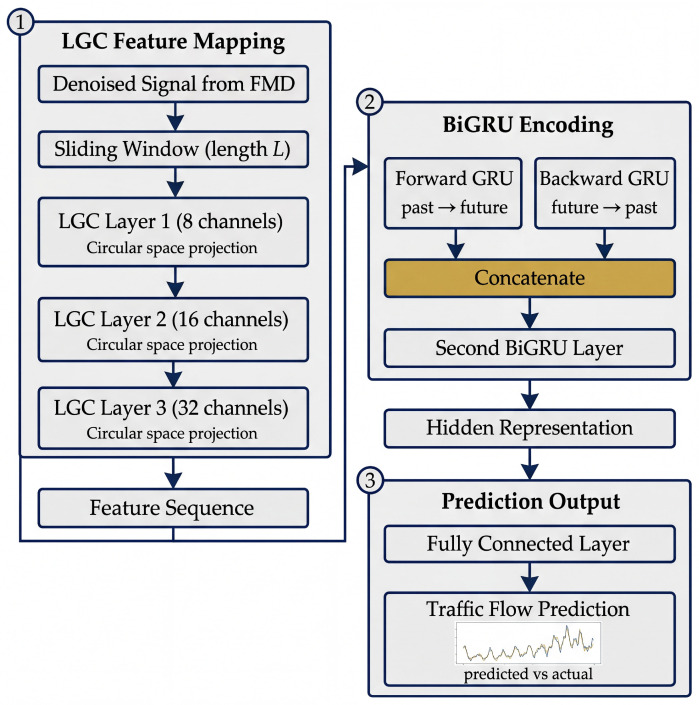
Operation flow of the LGC-BiGRU module.

As shown in Figure 4, the LGC-BiGRU module converts the denoised traffic signal into a future prediction through three consecutive stages: LGC feature mapping, BiGRU bidirectional coding, and fully connected regression.

LGC feature mapping: The module first receives the denoised signal $\hat{s}\left(t\right)$ from the FMD module and organizes it into a lookback window of length $L:\left\{\hat{s}\left(t-L+1\right),\hat{s}\left(t-L+2\right),\ldots ,\hat{s}\left(t\right)\right\}$. This sequence is then passed through three stacked LGC layers with increasing channel dimensions *C*_1_ = 8, *C*_2_ = 16, and *C*_3_ = 32. The core idea of the LGC layer is to project traffic features onto a circular probability space. Traffic flow exhibits a distinct periodic pattern, which can be represented by a circle. The LGC layer uses a Gaussian kernel centered at a specific angle to enhance features that correspond to these periodic rhythms.

For a given input $u_{t}\in R^{C_{n}}$ at time *t*, the LGC layer computes:

(19) $$g_{t}=\sigma \left(W_{g}u_{t}+b_{g}\right),$$

(20) $$h_{t}=\tanh\left(W_{h}u_{t}+b_{h}\right),$$

where $g_{t}$ is a gating vector that controls which features to retain, $h_{t}$ is a transformed feature vector, $W_{g}$ and $W_{h}$ are weight matrices, $b_{g}$ and $b_{h}$ are biases, and *σ* is the sigmoid activation function.

The angle of the gated feature is then calculated as follows:

(21) $$\varphi_{t}=\arctan2\left(g_{t},2,g_{t},1\right),$$

Angle $\varphi_{t}$ captures the timing of the current traffic pattern within a cycle. To highlight the pattern consistent with the expected period, we applied a Gaussian kernel function:

(22) $$K_{t}=\exp\left(-\frac{{\left(\varphi_{t}-\mu_{\omega }\right)}^{2}}{2\psi_{\omega }^{2}}\right),$$

where $\mu_{\omega }$ and $\psi_{\omega }$ are learnable parameters representing the mean and standard deviation of the circular kernel.

When $\varphi_{t}$ is aligned with $\mu_{\omega }$, the weight $K_{t}$ is close to 1; as the deviation increases, the weight $k_{t}$ decreases to 0. Then, the LGC layer multiplies the transformed feature $h_{t}$ by this weight:

(23) $$v_{t}=h_{t}\cdot k_{t},$$

The $v_{t}$ amplifies the features appearing at expected periodic times and suppresses those appearing at unexpected times. After passing through three LGC layers with 8, 16, and 32 channels, the output is a feature sequence expressed as:

(24) $$V=\left[v_{1},v_{2},\ldots ,v_{L}\right]\in R^{L\times 32},$$

BiGRU bidirectional encoding: The feature sequence *V* is fed into two stacked BiGRU layers. A BiGRU consists of two parallel GRUs: one reads the sequence forward, and the other reads the sequence backward. This bidirectional structure enables the model to capture temporal dependencies from both directions. This is highly advantageous for traffic flow prediction, as current traffic conditions are influenced by previous and subsequent traffic conditions. Furthermore, the stacking of two BiGRU layers enables hierarchical feature learning: the first layer captures short-term fluctuations, while the second layer identifies long-term temporal dependencies. This design allows BiGRU to more effectively model traffic dynamics across multiple time scales. For the forward GRU at time step *t*, its update gate ${\vec{z}}_{t}$ is expressed as follows:

(25) $${\vec{z}}_{t}=\sigma \left(W_{z}v_{t}+U_{z}{\vec{p}}_{t-1}+b_{z}\right),$$

where $v_{t}$ is the current input from the LGC output, ${\vec{p}}_{t-1}$ is the previous forward hidden state, $W_{z}$ and $U_{z}$ are the input and recurrent weight matrices for the update gate, and $b_{z}$ is the bias.

The function of the reset gate ${\vec{k}}_{t}$ in GRU is to control the degree of ignoring the previous hidden state, which is expressed as:

(26) $${\vec{k}}_{t}=\sigma \left(W_{k}v_{t}+U_{k}{\vec{p}}_{t-1}+b_{k}\right),$$

where $W_{k}$ and $U_{k}$ are the input and recurrent weight matrices for the reset gate, and $b_{k}$ is its bias.

The hidden state of the GRU candidate incorporates new information after resetting the previous state:

(27) $${\vec{\tilde{p}}}_{t}=\tanh\left(W_{p}v_{t}+U_{p}\left({\vec{k}}_{t}⊙{\vec{p}}_{t-1}\right)+b_{p}\right),$$

where $b_{p}$ is the bias vector in the calculation of candidate hidden states.

Finally, the forward hidden state is updated as a weighted combination of the previous state and the candidate:

(28) $${\vec{p}}_{t}=\left(1-{\vec{z}}_{t}\right)⊙{\vec{p}}_{t-1}+{\vec{z}}_{t}⊙{\vec{\tilde{p}}}_{t},$$

The backward GRU computes ${\overset{\leftarrow }{p}}_{t}$ using the same formulas but processes the sequence in reverse order. The BiGRU output at time *t* is the concatenation of the forward and backward hidden states:

(29) $$q_{t}={\vec{p}}_{t}⊕{\overset{\leftarrow }{p}}_{t},$$

where ⊕ denotes vector concatenation. The second BiGRU layer takes $q_{t}$ as input and produces the final hidden representation:

(30) $$c_{t}=\mathrm{BiGRU}\left(q_{t}\right),$$

where $c_{t}$ is the context vector at time *t*, encapsulating bidirectional temporal information from the entire sequence.

The final hidden state $c_{L}$ at the last time step of the lookback window is passed through a fully connected layer to generate the prediction:

(31) $$y_{\mathrm{output}}=W_{o}c_{L}+b_{o},$$

where $W_{o}$ is the weight matrix of the output layer, and $b_{o}$ is the bias term of the output layer.

The hyperparameters of this module—learning rate *ξ*, number of hidden units *H*, and regularization coefficient τ—do not need to be set manually. They are automatically optimized by the EBWO module using the prediction error as the fitness function, a process described as follows:

(32) $$\left(\xi^{*},H^{*},\tau^{*}\right)=\arg\underset{\xi ,H,\tau }{\min}\mathrm{MSE}\left(\xi ,H,\tau \right),$$

where $\mathrm{MSE}\left(\xi ,H,\tau \right)$ is the mean squared error of the LGC-BiGRU model on the training set under hyperparameters $\left(\xi ,H,\tau \right)$.

At this point, the entire prediction framework has completed a closed loop: FMD optimizes the raw sensor data, EBWO automatically optimizes the model hyperparameters, and LGC-BiGRU generates the final prediction results.

## 3. Results

This section reports in detail the experimental results of the proposed FMD-EBWO-LGC-BiGRU framework on four real-world traffic datasets. First, the experimental setup is introduced, followed by prediction examples, overall prediction performance comparisons, ablation experiments, EBWO optimizer comparisons, and a visualization analysis of FMD denoising.

### 3.1. Experiment Setup

To comprehensively evaluate the performance of the proposed FMD-EBWO-LGC-BiGRU framework, this paper uses the UTD19 traffic flow database (https://www.research-collection.ethz.ch/handle/20.500.11850/437802, accessed on 10 June 2026) for validation. This database covers different cities, different sampling frequencies, and different road types. Table 1 summarizes the detailed statistics for each dataset.

The reasons for selecting the detailed data used in the experiment from the dataset are as follows:

- For the Paris dataset, we selected data from sensor No. 533 in May to evaluate the model’s ability to predict macroscopic traffic flow at low temporal resolution. This sensor dataset exhibits gradual changes over a long period, allowing us to test the model’s ability to capture long-term dependencies and its resistance to underfitting.
- For the Rotterdam dataset, we selected sensor data from 30 October (No. 1132). This data was designed to test the model’s ability to simulate short- to medium-term temporal dependencies, particularly capturing dynamic changes during the transition between morning and evening rush hours.
- For the Berlin dataset, we used high-frequency sampling data from sensor K070 on 24 October. These traffic flow data exhibit instantaneous fluctuations and minor sudden events, aiming to test the model’s ability to suppress high-frequency noise and its response speed to short-term abrupt changes.
- We also selected high-frequency sampling data from sensor No. 202 in London on 27 September for cross-city comparison with the Berlin data under different road layouts. This further evaluates the model’s migration and generalization ability under diverse traffic flow characteristics, specifically its robustness against non-stationary noise and outliers in complex road networks.

In all prediction experiments, the lookback window length was set to 12 time steps to predict traffic flow at the next time step. Data were randomly divided into training, validation, and test sets in a 6:2:2 ratio [29,30]. For missing values, linear interpolation was used to fill in gaps for up to three consecutive missing time steps. All input data were normalized to the range [0, 1] using a min–maximum normalization method. The normalization parameters were calculated from the training set and applied to the validation and test sets to prevent data leakage.

To ensure reproducibility of prediction results and facilitate fair comparison, all key hyperparameters of the proposed FMD-EBWO-LGC-BiGRU model are fixed according to the values listed in Table 2. FMD module parameters are determined based on the signal characteristics of each dataset. EBWO parameters are set after preliminary sensitivity analysis, balancing convergence speed and population diversity. LGC-BiGRU network architecture parameters are adaptively optimized by EBWO within a specified range.

To evaluate the sensitivity of the proposed framework to key hyperparameter settings, we analyzed the impact of the number of BiGRU hidden units *H* and the learning rate *ξ* on model performance. Keeping other parameters constant, we varied these hyperparameters within the search range ($H\in \left[16,128\right],\;\xi \in \left[1\times 10^{-4},1\times 10^{-2}\right]$) using a validation set of 533 sensors. The results show that model performance is relatively stable when *H* is between 48 and 96, with MAE values fluctuating within ±3.5% of the optimal configuration. Extreme values (*H* = 16 or *H* = 128) lead to underfitting or overfitting, increasing MAE by 12–18%. For the learning rate, *ξ* = 1 × 10^−3^ achieves the best balance between convergence speed and final accuracy; *ξ* = 1 × 10^−4^ slows down convergence; and *ξ* = 1 × 10^−2^ leads to training instability. These results confirm that EBWO can effectively identify near-optimal configurations in the search space, thereby reducing the risk of performance degradation caused by manual configuration selection.

Based on the parameter configurations listed in Table 2, all models were implemented using MATLAB R2021a on a standard desktop computer (Intel Core i5 processor, 16 GB memory). To comprehensively evaluate the proposed FMD-EBWO-LGC-BiGRU framework, we selected six baseline and state-of-the-art models. The specific descriptions of each model are as follows:

- BiGRU: As a recurrent baseline model, used to validate the predictive advantages brought by bidirectional temporal information.
- VMD-BiGRU: As a decomposition-aided baseline model, utilizing VMD for denoising. It is directly compared with our FMD module.
- DCRNN (Diffusion Convolutional Recurrent Neural Network): A diffusing convolutional recurrent neural network that models traffic flow as a diffusion process on a directed graph [31]. It is used to evaluate the spatial dependency capture capability of our framework by comparing it with explicit spatial correlation modeling.
- STGCN (Spatiotemporal Graph Convolutional Network): A spatiotemporal graph convolutional network that uses graph convolution to handle spatial dependencies and temporal convolution to capture dynamic changes [32]. It is used to evaluate the accuracy of our framework’s prediction results.
- VMD-CNN-Transformer: This model first performs VMD on traffic signals and then uses a CNN-Transformer architecture for prediction [33]. It is used to validate the superiority of our framework over traditional quadratic decomposition and attention-based prediction methods.
- HSTGNN (Hybrid Spatiotemporal Graph Neural Network): A hybrid spatiotemporal graph neural network incorporating attention mechanisms. It combines static adaptive graph learning, dynamic graph learning, and attention mechanisms to model complex spatiotemporal relationships in road networks within a unified framework. Its prediction accuracy surpasses that of existing mainstream methods on multiple benchmark datasets, making it suitable for testing the ability of the FMD denoising model under this framework to capture and generalize complex spatiotemporal dynamics.

The above six models cover three key aspects: bidirectional temporal dependence (BiGRU, VMD-BiGRU), spatial and long-range dynamics (DCRNN, STGCN, HSTGNN), and decomposition-assisted denoising (VMD-BiGRU, VMD-CNN-Transformer). Fair comparisons under the same experimental settings will fully demonstrate the superior performance and generalization ability of the proposed framework.

To quantitatively evaluate the predictive performance of all models, we employed three standard metrics: Mean Absolute Error (MAE), Root Mean Square Error (RMSE), and Mean Absolute Percentage Error (MAPE). MAE measures the average deviation between predicted and actual traffic flow, assessing overall prediction accuracy [34]. Since traffic flow data typically has minimal measurement noise and few extreme outliers, MAE provides a stable and robust evaluation method [35]. RMSE reflects the model’s ability to avoid large errors [36]. Because RMSE squares the prediction error before averaging, it assigns higher weight to larger errors [37]. MAPE expresses the error as a percentage of actual traffic volume. It is scale-independent, allowing for fair comparisons across different sensors, cities, and traffic conditions [38,39]. Together, these three metrics reflect the model’s ability to capture traffic patterns and its consistency across different traffic scales.

### 3.2. Predictive Experiments and Analysis

**Figure 5:**
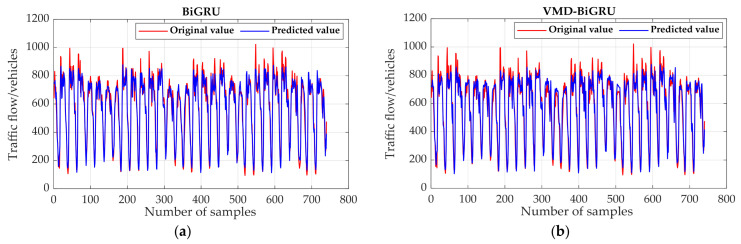

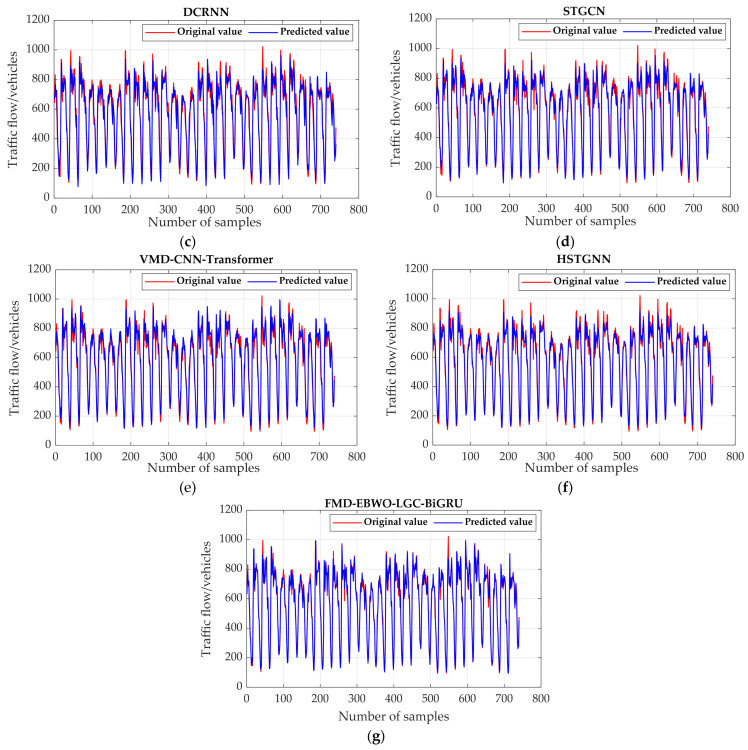
Prediction results of different models on the Paris dataset. (**a**) BiGRU prediction results; (**b**) VMD-BiGRU prediction results; (**c**) DCRNN prediction results; (**d**) STGCN prediction results; (**e**) VMD-CNN-Transformer prediction results; (**f**) HSTGNN prediction results; (**g**) FMD-EBWO-LGC-BiGRU prediction results.

To visually demonstrate the predictive power of the proposed FMD-EBWO-LGC-BiGRU framework, we first present the prediction results based on the Paris dataset (sensor No. 533, all data from May). Figure 5 shows a comparison between the traffic flow predicted by sensor No. 533 and the actual traffic flow.

As shown in Figure 5, the BiGRU model can capture the overall trend of traffic flow, but it exhibits significant lag during both morning and evening peak hours and often underestimates peak traffic levels. While the VMD-BiGRU model reduces some high-frequency noise, it still cannot accurately capture sharp increases and decreases in traffic flow. The DCRNN and STGCN models, due to the incorporation of spatial information, produce relatively smooth predictions. However, their prediction accuracy is insufficient to support the real-time traffic monitoring needs of ITS. The VMD-CNN-Transformer model improves peak alignment, but its prediction accuracy is poor during off-peak hours. The HSTGNN model, as the best-performing comparative model, shows good fitting, but its predictions still have biases near the shoulder of peak hours. In contrast, the proposed FMD-EBWO-LGC-BiGRU model’s predictions are almost identical to the actual values. It accurately captures the time and magnitude of morning and evening peaks, as well as the transition between peaks. This indicates that the framework has strong denoising, periodic pattern enhancement, and hyperparameter optimization capabilities.

To further validate the model’s generalization ability across different cities and sampling frequencies, Table 3, Table 4 and Table 5 report the evaluation metrics of all six comparative models and our proposed FMD-EBWO-LGC-BiGRU framework on the remaining sensor test set. All results are taken as the average of twenty independent runs and presented as mean ± standard deviation. For statistical significance assessment, we performed paired *t*-tests for each sensor dataset and each metric. The resulting *p*-values ranged from [1.2 × 10^−5^, 3.8 × 10^−3^], all less than 0.05. To control for multiple comparison bias, we applied Bonferroni correction, setting the significance threshold to α_corrected = 0.05/72 ≈ 6.9 × 10^−4^. Even with this conservative correction, all *p*-values remained statistically significant, confirming that our method significantly improves upon all competing models.

The results in Table 3, Table 4 and Table 5 consistently show that the proposed FMD-EBWO-LGC-BiGRU framework exhibits superiority across all four sensors and all evaluation metrics. Compared to all of the contrasting models, the proposed framework significantly reduces MAE across all four sensors. More importantly, compared to the state-of-the-art model HSTGNN, the proposed framework achieves an average MAE reduction of 30.6% across the four sensors. RMSE and MAPE show similar trends. The FMD-EBWO-LGC-BiGRU framework reduces RMSE by 40.6%, 22.9%, 28.9%, and 27.3% across the four sensors compared to HSTGNN, with an average reduction of 29.9%. For MAPE, the proposed method improves upon HSTGNN by 40.9%, 23.4%, 29.5%, and 27.9%, respectively, with an average reduction of 30.4%.

Furthermore, the standard deviation of FMD-EBWO-LGC-BiGRU is consistently the smallest among all models, indicating that the hyperparameters optimized by EBWO produce stable prediction results under different random seeds. While the decomposition-assisted comparative models (VMD-BiGRU and VMD-CNN-Transformer) show good prediction accuracy, they still lag behind our FMD-based method. This confirms the advantage of the adaptive frequency domain segmentation mechanism over traditional mode decomposition methods. Although spatiotemporal graphical models (DCRNN, STGCN, HSTGNN) incorporate structural information, their performance is limited by the quality of the original input data. After FMD denoising, the proposed framework outperforms them without any explicit graphical convolutions. These quantitative results demonstrate the advantages of the proposed method in terms of prediction accuracy and robustness.

It is noteworthy that VMD-BiGRU and our FMD-based framework share the same BiGRU prediction architecture, allowing for a direct comparison of the decomposition methods. As shown in Table 3, Table 4 and Table 5, our framework achieves significant improvements over VMD-BiGRU: a 67.5% reduction in MAE for sensor No. 533, a 66.6% reduction for sensor No. 1132, a 64.2% reduction for sensor K070, and a 67.1% reduction for sensor No. 202. These consistent and significant improvements strongly demonstrate the advantages of the FMD adaptive frequency domain segmentation mechanism over traditional VMD. Although spatiotemporal models (DCRNN, STGCN, HSTGNN) incorporate spatial information, their performance still lags behind our framework, which uses only temporal information. This finding highlights that data quality is far more important than model complexity. Furthermore, our framework also significantly outperforms VMD-CNN-Transformer (MAE reductions of 41.2%, 24.2%, 32.0%, and 28.9% for the four sensors, respectively). This confirms that the superiority of our method stems not solely from the choice of predictor, but from the effectiveness of the FMD module.

### 3.3. Ablation Study

To systematically evaluate the contribution of each key component in the proposed FMD-EBWO-LGC-BiGRU framework, we constructed four variants for comprehensive ablation studies:

- w/o FMD: Raw sensor data bypasses the FMD module and is directly input into the LGC-BiGRU prediction module. The EBWO and LGC modules remain unchanged.
- w/o EBWO: The hyperparameters of the LGC-BiGRU module are fixed via grid search (learning rate = 0.001, number of hidden units = 64, regularization strength = 5 × 10^−4^). The FMD and LGC modules remain active.
- w/o LGC: The three Logistic-Gauss Circle layers are replaced with standard linear layers with the same input/output dimensions. The FMD and EBWO modules remain operational.
- Full model: The FMD-EBWO-LGC-BiGRU framework containing all three modules.

Each variant was trained and evaluated five times under the same experimental conditions using different random seeds. Table 6 lists the average MAE, RMSE, and MAPE results for each sensor across twenty runs.

Impact of FMD: Removing the FMD module significantly reduces the prediction accuracy of all sensor data. Specifically, the MAE increased by 128.4% for sensor No. 533, 122.4% for sensor No. 1132, 143.1% for sensor K070, and 129.9% for sensor No. 202. These large error jumps clearly indicate that the raw sensor data contains a large amount of non-stationary noise and outliers, severely misleading the EBWO-LGC-BiGRU module. Although the LGC-BiGRU model can capture bidirectional time dependencies, it cannot distinguish between real traffic patterns and high-frequency noise spikes. The FMD module effectively cleanses the input signal by adaptively partitioning the frequency domain while preserving crucial traffic dynamics information.

Impact of EBWO: Removing EBWO continuously reduces the prediction accuracy of all sensor data. Compared to the complete framework, the MAE increased by 43.6%, 44.9%, 43.2%, and 46.1% for No. 533, No. 1132, K070, and No. 202, respectively. Manually set parameters are not optimal for any dataset. For example, on the high-frequency Berlin data (K070), EBWO found that 112 hidden units were needed to capture complex temporal variations, while the manually selected 64 units were far from sufficient. Therefore, automatic hyperparameter optimization using EBWO is crucial for achieving high prediction accuracy, especially when data characteristics differ across cities.

Impact of LGC: Compared to the full model, removing the LGC layer leads to a 17.2–21.5% increase in MAE. The increase in MAPE is even greater (up to 27.9% in sensor No. 533). This indicates that LGC does indeed contribute to improving prediction accuracy, especially in terms of relative error. This improvement stems from a Gaussian kernel centered on a learnable angle, which amplifies features consistent with the expected diurnal cycle and suppresses features occurring in aperiodic periods.

Ablation experiments show that each module plays a unique and indispensable role. FMD contributes the most, increasing MAE by 122.4% to 143.1% after removal, confirming that denoising is the most critical preprocessing step. EBWO has the second largest impact, increasing MAE by 43.2% to 46.1% after manual hyperparameter tuning. LGC provides a smaller but still significant contribution (increasing MAE by 17.2% to 21.5%), confirming its role in enhancing sensitivity to periodic patterns. These results collectively indicate that these three modules are not redundant but rather work synergistically, each playing a role in different aspects of the traffic flow prediction challenge.

### 3.4. Benchmarking EBWO with Other Optimizers

**Figure 6:**
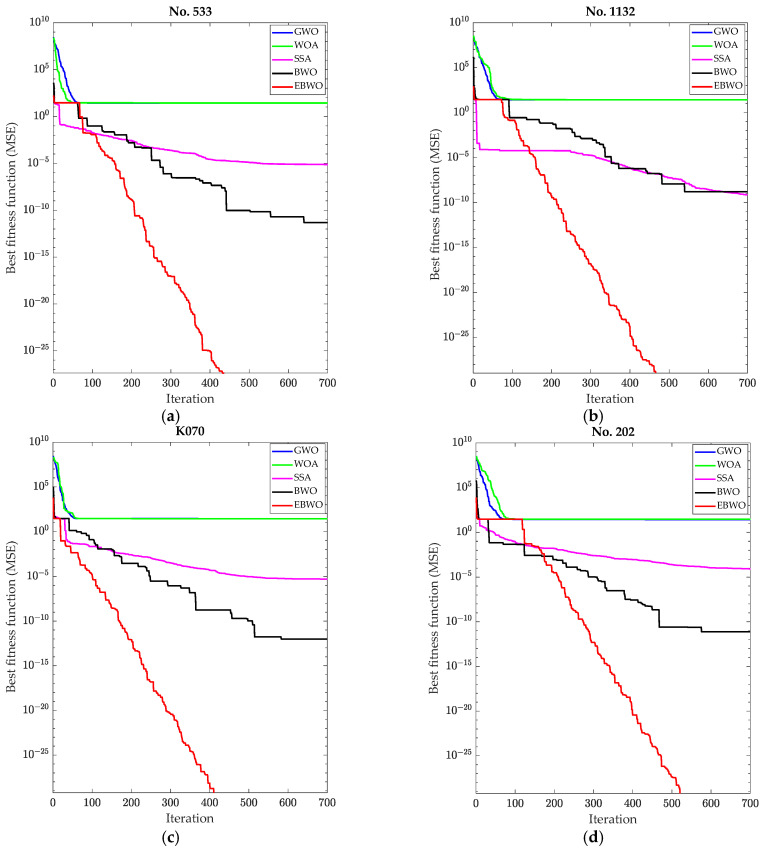
The convergence performance and speed of different optimizers. (**a**) Convergence results of different optimizers in No. 533; (**b**) Convergence results of different optimizers in No. 1132; (**c**) Convergence results of different optimizers in K070; (**d**) Convergence results of different optimizers in No. 202.

To verify the superiority of the proposed EBWO, we compared it with four advanced metaheuristic optimizers: Grey Wolf Optimization (GWO) [40], Whale Optimization (WOA) [41], Sparrow Search Algorithm (SSA) [42], and the original Beluga Whale Optimization (BWO) [43]. All optimizers performed hyperparameter searches on four sensor datasets of the LGC-BiGRU model. To ensure fairness, each optimizer was run independently 20 times, with the same population size (30) and maximum number of iterations (700) for each run. Since mean squared error (MSE) effectively suppresses sporadic large errors, guiding the optimizer to achieve more robust real-time performance, we used it as the evaluation metric [44,45]. Figure 6 shows the optimal convergence performance and speed of different optimizers on different sensors.

As shown in Figure 6, the convergence curves reveal a steep decline in EBWO’s fitness value during the initial iterations, indicating its ability to rapidly approach the optimal solution. This is primarily attributed to the population diversity introduced by the chaotic initialization of Tent, which allows the algorithm to broadly cover the hyperparameter space during the early exploration phase. Between iterations 15 and 25, the fitness value of EBWO gradually decreases, reaching a stable state after approximately 25 iterations. In contrast, the convergence speeds of GWO and WOA are significantly slower: GWO only begins to approach a plateau after 70 iterations; although WOA accelerates slightly in the middle stage, it still requires about 60 iterations to reach an error level similar to EBWO. This indicates that the balance factor design of GWO and WOA is relatively fixed and cannot dynamically adjust the ratio of exploration to development according to the search progress. The curves of SSA and BWO show obvious sawtooth fluctuations, and oscillations still exist even after 400 iterations, indicating their poor stability in hyperparameter optimization problems. This is because their position update mechanism is too random, making it difficult to maintain consistency in the fine-grained search phase.

Table 7 reports the average runtime required for each optimizer to complete the optimization in the experiment.

To assess the feasibility of deploying this framework in practice, we analyze its computational cost from two aspects: optimization time and inference efficiency. According to Table 7, EBWO exhibits the fastest convergence speed across all four sensors. For example, using sensor No. 533, EBWO takes 7.6 s, while GWO, WOA, SSA, and BWO take 29.6 s, 24.2 s, 38.5 s, and 17.8 s, respectively. EBWO also significantly outperforms other optimizers on other sensors. In summary, EBWO achieves the fastest convergence speed while maintaining optimization quality. This makes it an ideal choice for our prediction framework configuration.

Besides optimization time, the inference efficiency of the trained model is also crucial for real-time ITS applications. On our test hardware (Intel Core i5 processor, 16 GB memory), the average inference time per prediction is 35 milliseconds for the low-frequency Paris dataset and 43 milliseconds for the high-frequency Berlin dataset. These data fully meet the requirements of real-time applications, with inference latency typically below 100 milliseconds for traffic signal control and below 50 milliseconds for dynamic path guidance. The trained LGC-BiGRU model has a memory footprint of approximately 45 MB, and the filter bank of the FMD module requires an additional 12 MB, totaling approximately 57 MB. This lightweight memory requirement makes the framework well-suited for deployment on resource-constrained edge devices.

### 3.5. Denoising Visualization of FMD

**Figure 7:**
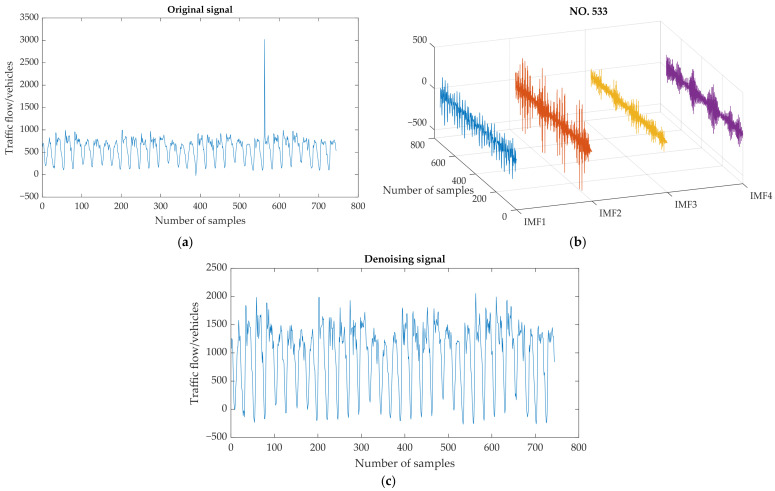
Decomposition results of data signal from sensor NO. 533. (**a**) Original signal No. 533; (**b**) FMD signal decomposition results; (**c**) Signal after denoising.

To visually demonstrate the denoising capability of the FMD module, we provided a decomposition example using data from sensor No. 533 (Figure 7). All FMD parameters are the same as those used in the prediction experiments.

As shown in Figure 7a, the original signal exhibits significant high-frequency fluctuations and two outliers. Figure 7b shows the IMFs extracted from the original signal. Adding the individual IMFs yields the denoised signal shown in Figure 7c. Compared to the original signal, the denoised signal in Figure 7c is smoother, accurately preserving the time and amplitude of morning and evening peak hours, and effectively correcting outliers. The decomposition results of the other three sensors (No. 1132, K070, and No. 202) also show similar patterns. Therefore, the FMD module designed in this paper does not require a preset number of modes and can effectively separate the main traffic pattern from noise.

**Figure 8:**
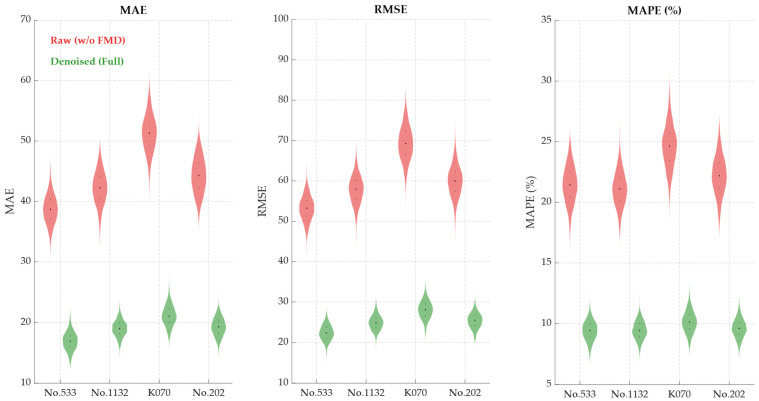
Prediction error plots of the original data and FMD-denoised data.

To further quantify the denoising effect of the FMD module and compare the prediction error distributions of the original data (without FMD application) and the FMD denoising model (complete framework), we generated violin plots based on statistical parameters obtained from ablation experiments. For each sensor and each error metric, we assumed that the error follows a normal distribution and used the corresponding empirical mean and standard deviation to simulate a large number of error samples. Figure 8 shows the prediction error violin plots for the four sensors.

As shown in Figure 8, the green portion of each sensor and each index is significantly lower than the red portion, confirming that FMD significantly reduces the central tendency of prediction errors. This improvement is consistent with the quantitative results reported in the ablation study. Secondly, the distribution width of the green portion is generally smaller than that of the red portion, reflecting that FMD improves the stability of prediction results while reducing error dispersion. Furthermore, the error distribution of the original data exhibits a significant right-skewed characteristic, while after FMD processing, the error distribution tends to be symmetrical and more compact, confirming that the adaptive frequency domain segmentation mechanism can effectively suppress prediction fluctuations caused by outliers. The above visual evidence fully demonstrates that, regardless of the sampling interval or urban traffic patterns, FMD can consistently improve the accuracy and robustness of traffic flow prediction.

## 4. Discussion

The proposed FMD-EBWO-LGC-BiGRU framework offers several practical advantages for real-world ITS deployments. This framework is particularly suitable for real-time traffic signal control, where accurate short-term forecasts enable adaptive signal timing adjustments, thereby alleviating traffic congestion. It is also applicable to dynamic route guidance systems, where reliable traffic forecasts provide proactive route reselection suggestions to road users. Furthermore, the framework is suitable for edge-based traffic monitoring, and its time-only design reduces the computational burden on resource-constrained devices.

Despite the excellent performance of the framework, it still has some inherent limitations. The most significant limitation lies in its time-only design: the framework does not explicitly model the spatial correlations between adjacent sensors. Although this design reduces complexity and enables lightweight deployment, it also means that the framework cannot leverage information from neighboring sensors to improve prediction accuracy, especially when individual sensors fail or provide unreliable data. In addition, FMD relies on the discrete Fourier transform, which suffers from low-frequency resolution over very short time windows, potentially affecting the accuracy of frequency band detection in such scenarios. Regarding data completeness, we have not systematically evaluated the robustness of the framework under various missing-data scenarios, such as different missing rates or consecutive missing patterns. The “w/o FMD” variant in the ablation study indirectly suggests that the model is sensitive to degraded data quality, thus calling for more comprehensive investigations. Finally, although our experiments cover sensors from multiple cities, sampling frequencies, and road types, we have not explicitly assessed the model’s robustness to variations in road segment length, road capacity, signal timing loss, or spillover effects. This is because the UTD19 database does not provide detailed infrastructure or signal-timing information. Therefore, evaluating the framework using a professional traffic simulation platform remains an important direction for future research.

To address the above limitations, we plan to pursue several future research directions. The most important direction is to extend the current time-only framework into a spatiotemporal model by integrating graph neural networks. This will enable the model to explicitly capture spatial correlations between adjacent sensors and is expected to further improve prediction accuracy at the road network scale, thereby solving the challenge of network-level multi-sensor prediction. To reduce the optimization burden when scaling up to hundreds of sensors, we will develop a transfer learning mechanism that can share optimized hyperparameters among sensors with similar traffic patterns, thus reducing the number of training iterations required for EBWO in multi-sensor deployments. We will also design an adaptive periodic detection module combined with an attention mechanism to dynamically identify non-standard periodic fluctuations that occur during anomalous periods such as special events and extreme weather, thereby improving the robustness of the LGC module. To meet the real-time deployment requirements on edge devices, we will explore model compression techniques, including knowledge distillation and weight quantization, which are expected to reduce inference latency by 20–30% and memory footprint by 50%. We also plan to use the SUMO and VISSIM traffic simulation platforms to systematically evaluate the framework’s robustness under varying road segment lengths, road capacities, signal timing, and spillover effects. Finally, we will conduct in-depth studies on how different missing-data patterns affect prediction accuracy, aiming to develop adaptive data imputation strategies that can cope with sensor failures. These research directions will help extend the proposed framework to a broader range of intelligent transportation applications.

## 5. Conclusions

This paper proposes a hybrid traffic flow prediction framework, FMD-EBWO-LGC-BiGRU. This framework effectively addresses the challenges of sensor data noise and hyperparameter tuning. FMD eliminates mode aliasing and adaptively separates noise from traffic signals without pre-setting the number of modes. EBWO accelerates convergence and improves solution quality through chaotic initialization, adaptive balancing factors, and an improved Lévy algorithm. LGC-BiGRU enhances sensitivity to periodic modes through circular projection and captures bidirectional time dependencies. Experiments on four real-world datasets with different sampling intervals demonstrate that the proposed framework consistently outperforms six comparative models. Ablation experiments and convergence experiments confirm the indispensable contributions of each module and the efficiency of EBWO. In summary, this framework provides a solution for traffic flow prediction in resource-constrained ITS environments that combines accuracy and efficiency.

## Figures and Tables

**Table 1:** Dataset statistics.

City	Sampling Interval	Sensors	Time Range
Paris	1 h	67	March 2016–November 2016
Rotterdam	15 min	92	September 2017–November 2017
Berlin	5 min	120	October 2016–December 2016
London	5 min	80	May 2015–September 2015

**Table 2:** Key model parameter settings.

Parameter	Value/Range
Standard deviation *σ*	2.0
Energy threshold *ε*	0.05
High-frequency cutoff *f*_high_	10 Hz (for all datasets)
Tent chaos parameter *β*	0.5
Maximum generations *G*_max_	120
Lévy exponent *λ*	1.5
Initial step size α_0_	0.01
Diversity threshold Div^(*g*)^	0.045
Mean of the circular kernel $\mu_{\omega }$	[0, 2π]
Standard deviation of the circular kernel $\psi_{\omega }$	[π/12, π/2]
Learning rate search range *ξ*	[1 × 10^−4^, 1 × 10^−2^]
BiGRU hidden cell search range *H*	[16, 128] (integer)
Regularization coefficient *τ*	[1 × 10^−6^, 1 × 10^−3^]
EBWO population size	700
Optimizer (inner loop)	Adam
Early stopping	20
Maximum epochs	100 (per fitness evaluation)

**Table 3:** MAE results for predictions from different models.

Model	No. 533	No. 1132	K070	No. 202
BiGRU	62.150 ± 2.13	65.037 ± 1.82	72.119 ± 2.51	67.205 ± 2.27
VMD-BiGRU	50.253 ± 1.96	54.521 ± 1.64	57.337 ± 2.18	56.184 ± 1.93
DCRNN	44.091 ± 2.05	43.300 ± 1.77	46.720 ± 2.34	46.039 ± 2.01
STGCN	35.163 ± 1.88	35.047 ± 1.55	38.015 ± 2.21	35.170 ± 1.96
VMD-CNN-Transformer	27.911 ± 1.73	24.032 ± 1.42	30.149 ± 2.07	26.013 ± 1.84
HSTGNN	27.835 ± 1.68	24.026 ± 1.51	28.913 ± 1.95	25.730 ± 1.77
FMD-EBWO-LGC-BiGRU	16.410 ± 1.52	18.209 ± 1.34	20.507 ± 1.86	18.505 ± 1.63

**Table 4:** RMSE results for predictions from different models.

Model	No. 533	No. 1132	K070	No. 202
BiGRU	79.240 ± 2.68	83.510 ± 2.31	94.106 ± 3.14	86.705 ± 2.82
VMD-BiGRU	64.914 ± 2.45	70.472 ± 2.07	74.808 ± 2.73	72.908 ± 2.49
DCRNN	57.495 ± 2.57	56.622 ± 2.21	61.304 ± 2.96	60.226 ± 2.63
STGCN	46.820 ± 2.34	45.930 ± 1.98	49.915 ± 2.85	46.280 ± 2.47
VMD-CNN-Transformer	37.927 ± 2.16	35.277 ± 1.83	40.193 ± 2.58	34.525 ± 2.25
HSTGNN	36.533 ± 2.08	31.281 ± 1.94	38.570 ± 2.47	34.104 ± 2.18
FMD-EBWO-LGC-BiGRU	21.704 ± 1.94	24.119 ± 1.67	27.405 ± 2.28	24.801 ± 1.96

**Table 5:** MAPE (%) results for predictions from different models.

Model	No. 533	No. 1132	K070	No. 202
BiGRU	31.128 ± 1.34	30.316 ± 1.07	32.830 ± 1.42	30.522 ± 1.22
VMD-BiGRU	25.140 ± 1.21	25.450 ± 0.96	26.077 ± 1.28	25.517 ± 1.14
DCRNN	22.300 ± 1.25	20.274 ± 1.05	21.212 ± 1.33	20.909 ± 1.18
STGCN	17.604 ± 1.12	16.328 ± 0.94	17.335 ± 1.21	16.059 ± 1.05
VMD-CNN-Transformer	14.085 ± 1.03	11.236 ± 0.86	13.703 ± 1.12	11.824 ± 0.98
HSTGNN	13.900 ± 0.98	11.208 ± 0.82	13.195 ± 1.05	11.711 ± 1.94
FMD-EBWO-LGC-BiGRU	8.220 ± 0.82	8.590 ± 0.78	9.308 ± 0.93	8.441 ± 0.64

**Table 6:** Ablation experiment results based on data from different sensors.

Sensor	Variant	MAE	RMSE	MAPE (%)
No. 533	w/o FMD	38.67 ± 2.45	53.12 ± 3.21	21.48 ± 1.52
	w/o EBWO	24.31 ± 1.88	32.05 ± 2.34	13.51 ± 1.08
	w/o LGC	19.84 ± 1.62	26.78 ± 1.95	11.02 ± 0.93
	Full model	16.93 ± 1.48	22.41 ± 1.86	9.41 ± 0.78
No. 1132	w/o FMD	42.15 ± 2.67	57.83 ± 3.45	21.08 ± 1.42
	w/o EBWO	27.46 ± 2.01	36.22 ± 2.56	13.73 ± 1.12
	w/o LGC	22.57 ± 1.73	29.98 ± 2.12	11.29 ± 0.96
	Full model	18.95 ± 1.41	24.89 ± 1.78	9.48 ± 0.73
K070	w/o FMD	51.24 ± 3.08	69.36 ± 4.12	24.62 ± 1.68
	w/o EBWO	30.18 ± 2.24	40.15 ± 2.89	14.52 ± 1.21
	w/o LGC	24.89 ± 1.96	33.07 ± 2.46	11.96 ± 1.02
	Full model	21.08 ± 1.72	28.14 ± 2.15	10.14 ± 0.85
No. 202	w/o FMD	44.32 ± 2.83	60.05 ± 3.76	22.16 ± 1.56
	w/o EBWO	28.15 ± 2.11	37.08 ± 2.71	14.08 ± 1.18
	w/o LGC	23.41 ± 1.82	30.89 ± 2.31	11.71 ± 0.99
	Full model	19.27 ± 1.55	25.46 ± 1.92	9.64 ± 0.76

**Table 7:** Average completion time for hyperparameter optimization.

Optimizer	No. 533	No. 1132	K070	No. 202
GWO	29.6 s	30.8 s	33.2 s	27.7 s
WOA	24.2 s	26.5 s	29.3 s	25.1 s
SSA	38.5 s	38.9 s	40.7 s	37.2 s
BWO	17.8 s	19.4 s	18.8 s	18.9 s
EBWO	7.6 s	8.5 s	9.2 s	8.2 s

## Data Availability

The original contributions presented in this study are included in the article. Further inquiries can be directed to the corresponding author.

## Nomenclature

The following symbols are used in this manuscript:

*s*(*t*)	Potential clean traffic signals
*η*(*t*)	Non-stationary noise component
*δ*(*t*)	Sparse outlier component
*X*(*f*)	Fourier coefficient at frequency *f*
*P*(*f*)	Power spectral density
*m*	Integer offset for smoothing window
*g*(*m*)	Gaussian kernel
$\Omega_{i}$	FMD *i*-th frequency band
*Y_i_*(*f*)	Filter response of frequency band *i*
*H*_i_(*f*)	Filter response at frequency *f*
$r_{i}$	Energy contribution rate of the *i*-th mode
$f_{i}^{\mathrm{peak}}$	Peak frequency of the *i*-th mode
*ε*	Energy threshold for noise rejection
$f_{\mathrm{high}}$	High-frequency cutoff
*N*	Population size of EBWO
*D*	Number of hyperparameters to be optimized
*θ*	Hyperparameter configuration vector
*F*(*θ*)	Fitness function
$z_{p+1}$	Tent chaotic sequence state
$\theta_{d}^{\left(0\right)}$	Initial value of the *d*-th hyperparameter
*L_d_*	The lower bound of the d-th hyperparameter
*U_d_*	The upper bound of the d-th hyperparameter
*g*	Current iteration number
*G*_max_	Maximum number of iterations
*B*^(*g*)^	Adaptive balance factor at generation *g*
$\theta_{\mathrm{best}}$	Best configuration found so far
Lévy(*λ*)	Lévy flight step with exponent λ
*α*	Adaptive scaling factor
$\alpha_{0}$	Initial step size
Div^(*g*)^	Population diversity at generation *g*
*L*	Lookback window length
$v_{t}$	Input feature at time *t*
$g_{t}$	Gating vector
$z_{t}$	Transformed feature vector
$\varphi_{t}$	Phase angle of gated feature
*σ*	sigmoid activation function
$h_{t}$	Features after LGC layer transformation
$\mu_{\omega }$	Mean of the circular kernel
$\psi_{\omega }$	Standard deviation of the circular kernel
$v_{t}$	LGC layer output
${\vec{z}}_{t}$	In the forward GRU layer at time step *t*
${\vec{p}}_{t-1}$	The previous forward hidden state of GRU
$W_{z}$	Input weight matrix of GRU update gate
$U_{z}$	Circular weight matrix of GRU update gate
${\vec{k}}_{t}$	Reset gate in GRU
$W_{k}$	Input weight matrix of the reset gate
$U_{k}$	Circular weight matrix of the reset gate
$q_{t}$	BiGRU connection between forward and backward hidden states
$c_{L}$	GRU final hidden state
$c_{t}$	Final context vector
$W_{o}$	Weight matrix of GRU output layer
$y_{\mathrm{output}}$	Predicted traffic flow
*ξ*	Learning rate search range
*H*	Number of BiGRU hidden units
*τ*	Regularization coefficient

## Abbreviations

The following abbreviations are used in this manuscript:

ITS	Intelligent Transportation System
FMD	Frequency Mode Decomposition
EBWO	Enhanced Beluga Whale Optimization
LGC	Logistic Gaussian Circle
BiGRU	Bidirectional Gated Recurrent Unit
BiLSTM	Bidirectional Long Short-Term Memory
EMD	Empirical Mode Decomposition
VMD	Variational Mode Decomposition
CNN	Convolutional Neural Network
GRU	Gated Recurrent Unit
HSTGNN	Hybrid Spatiotemporal Graph Neural Network
IMF	Intrinsic Mode Function
DCRNN	Diffusion Convolutional Recurrent Neural Network
STGCN	Spatiotemporal Graph Convolutional Network
GWO	Grey Wolf Optimization
WOA	Whale Optimization Algorithm
SSA	Sparrow Search Algorithm
BWO	Beluga Whale Optimization
MAE	Mean Absolute Error
RMSE	Root Mean Square Error
MAPE	Mean Absolute Percentage Error
MSE	Mean Squared Error
